# Body-Temperature Programmable Soft-Shape Memory Hybrid Sponges for Comfort Fitting

**DOI:** 10.3390/polym13203501

**Published:** 2021-10-12

**Authors:** Balasundaram Selvan Naveen, Azharuddin Bin Mohamed Naseem, Catherine Jia Lin Ng, Jun Wei Chan, Rayner Zheng Xian Lee, Leonard Ee Tong Teo, Taoxi Wang, Mathews Nripan, Wei Min Huang

**Affiliations:** 1 School of Mechanical and Aerospace Engineering, Nanyang Technological University, 50 Nanyang Avenue, Singapore 639798, Singapore; naveen008@e.ntu.edu.sg (B.S.N.); azha0007@e.ntu.edu.sg (A.B.M.N.); cath0010@e.ntu.edu.sg (C.J.L.N.); jchan068@e.ntu.edu.sg (J.W.C.); rlee035@e.ntu.edu.sg (R.Z.X.L.); lteo012@e.ntu.edu.sg (L.E.T.T.); 2 College of Aerospace Engineering, Nanjing University of Aeronautics and Astronautics, 29 Yudao Street, Nanjing 210016, China; wa0003xi@nuaa.edu.cn; 3 School of Materials Science & Engineering, Nanyang Technological University, 50 Nanyang Avenue, Singapore 639798, Singapore; nripan@ntu.edu.sg

**Keywords:** shape memory hybrids, sponge, comfort fitting, programming, body temperature, recovery, hydrogel, lightweight, shape fixity ratio, shape recovery ratio, shape memory cycle

## Abstract

Porous shape memory hybrids are fabricated with different matrix (silicone) hardness and different inclusion (polycaprolactone, PCL) ratios. They are characterized to obtain their mechanical response to cyclic loads (with/without pre-straining/programming) and their shape memory performances after body-temperature programming are investigated. These materials are lightweight due to their porous structures. Wetted hydrogels used in the fabrication process for creating pores are reusable and hence this process is eco-friendly. These porous shape memory hybrids exhibit the good shape memory effect of around 90% with higher inclusion (PCL) ratios, which is better than the solid versions reported in the literature. Hence, it is concluded that these materials have great potential to be used in, for instance, insoles and soles for comfort fitting, as demonstrated.

## 1. Introduction

Customization has been the norm of the 21st century, particularly in the textile and footwear industries. More and more people prefer to have products that are personalised to fit them exactly. Hence, comfort fitting has taken a greater significance in products such as footwear, clothing, etc. Customised insoles provide greater comfort to users during walking and running exercise, etc. [1]. Customized footwear also helps to minimize the risk of injuries caused by standard footwear that do not fit conformably, according to the foot contour of the user. Moreover, orthotic devices, either fully customised or semi-customised, are extremely helpful in preventing lower extremity injuries during walking [2,3,4].

In recent years, 3D printing has been gaining significance in the field of manufacturing [5,6,7]. More and more importance has been given to the development of customizable footwear to improve comfort levels while wearing and also to prevent the occurrence of injuries due to misfit of standardised footwear. Many new techniques have been tried out in the past few years to some good outcomes, the most popular of which is the use of 3D printing technology to print footwear according to the scanned images of a user’s foot [8,9,10,11,12]. TPU/encapsulated paraffin blends were fabricated by 3D printing to explore the possibility of using them as sole materials in shoes [13]. However, acquiring 3D scanned images of a user’s foot and the 3D printing machines are still proving to be extremely expensive. Hence, the products developed through this technology are still not affordable for common people, coupled with the disadvantage of not being able to reshape once produced.

Shape memory polymers (SMP) could be a possible solution to these problems. They have started to attract a lot of attention in this field, as they have the unique potential to be programmed or reshaped even after fabrication, with the help of stimuli such as heat (thermo-responsive), light (photo-responsive) and chemical (chemo-responsive) [14,15,16,17,18,19,20,21]. In addition to that, the elastic version of SMP is not only bendable, but also stretchable. These properties enable them to be used in a wide variety of applications, ranging from actuator assemblies to biomedical applications [22,23,24,25,26], which make them very good candidates for producing customisable footwear and orthotic devices.

The unique feature associated with SMPs is called the shape memory effect (SME), i.e., an SMP material recovers its original shape only when exposed to a certain stimuli such as heat, light, chemicals, moisture, etc. [27,28]. Utilizing this feature, shape memory foams made from polyurethane (PU) and ethylene vinyl acetate (EVA) have already been produced commercially to be used as insoles [29,30,31]. The possibility of using density gradient polyurea foams was explored as well [32]. Thermoplastic PU (TPU) foams incorporated with thermoplastic amide elastomer (TPAE) was studied in [33]. PU is reported to have good heat responsive shape recovery when heated to high temperatures [34]. Thermo-responsive polymeric foam made from EVA can also be used as a shape memory insole by utilizing its heat responsive SME property to re-shape it according to the foot contours of a user [35].

Even though there are commercially available shape memory foams, they are activated (programable) only at high temperatures, which makes them uncomfortable to use for customisable footwear. This is because, in order to program the foam to a user’s foot, the foam is heated to high temperatures (well above the body temperature) and then fitted to the user’s foot mostly above the human body temperature. This process is not practical because the high temperatures will cause discomfort and sometimes even burns to the user’s foot. Hence, a material that can be programmed at body-temperature is essential for it to be used as a customisable footwear product.

Shape memory hybrid (SMH), a concept proposed in [36], is made of two components, the elastic component and the transition component, neither of which has the SME as an individual within the working environment. The major advantage of SMH is the convenience in design and fabrication to meet the requirements of a particular application [37]. This advantage is mainly due to the fact that we can select the components for an SMH from a wide range of materials to meet the required shape memory performance [38,39,40]. This property makes SMH an ideal material for the fabrication of customisable footwear.

Various elastic shape memory materials have been developed based on the principle of SMH [41,42,43]. The development of an elastic shape memory hybrid made with silicone elastomer, which is highly elastic (elastic component), and melting glue, which is the transition component, has been reported in [44]. According to the working principle revealed in [38], the elastic component in the SMH is always elastic within the working environment and stores the elastic energy during programming. The hard transition component in the SMH at room temperature softens when heated to above its transition (softening) temperature, so that it is easily deformable. Upon cooling, it is able to maintain the deformed shape once the transition component hardens. This is how the temporary shape is programmed in an SMH. Then, upon heating once again to above its transition temperature, the transition component softens again, thereby enabling the release of the stored elastic energy by the elastic component in the SMH. This helps in the shape recovery of the SMH. Melting glue used in [44] has higher crystallization temperatures of around 55 °C which makes it unsuitable for use as a comfort fitting material in contact with the human body. In [45], a different transition material, called PPM, which has PU and EVA as its main compositions, was used. They were able to achieve body temperature programming with this transition component and managed to get a shape fixity ratio of around 78%. However, an even higher shape fixity ratio is desirable particularly for comfort fitting purposes, as a lower shape fixity ratio would lead to a tight fit thereby causing discomfort to the user. In [44,45,46], polydimethylsiloxane (PDMS, SYLGARD^®^ 184) from Dow Corning Corporation (Midland, MI, USA) was applied as the elastic component. This material has a shore hardness of around 50A. In [47], thermoplastic elastomeric foams made from poly(styrene-(ethylene-co-butylene)-styrene (SEBS) were reported. However, the hardness achieved by them (40A) is still considered to be on the harder side.

For comfort fitting purpose in our daily life, such as shoes, the material needs to meet a series of requirements, including being soft, elastic, lightweight, and cheap, with enough time for fitting at body/room temperature.

Hence, in this study, various soft and porous SMHs are fabricated and their mechanical behaviour are characterised. Porosity is introduced by mixing wetted hydrogel in the fabrication process. These hydrogels are also reusable and eco-friendly, as evaporating the water from them recovers their original size and hence can be reused multiple times. In addition, the shape memory performance of these SMHs is investigated to analyse if they can be used as a potentially great alternative to the currently existing materials in the market for, for instance, soles. A shape memory shoe produced by using these soft and porous materials is also reported here for demonstration.

## 2. Materials, Sample Preparation and Experimental Procedure

### 2.1. Materials

The main materials used in this study are elastic silicone elastomers and polycaprolactone (PCL). The silicone elastomers were purchased from Shenzhen Hong Ye Jie Technology Co. Ltd. (Shenzhen, China). These types of silicones are highly stretchable and have a working temperature range from −65 °C to 200 °C. Two variants of this type of silicone elastomers (E610 and E640) were used, with the difference being that E610 has a Shore hardness of 10A, whereas E640 has a Shore hardness of 40A. Each silicone comes in two parts, namely Part A and Part B, both of which come in liquid form. They are recommended to be mixed in 1:1 volume ratio by the manufacturer. At room temperature, E610 silicone cures in about three hours, while E640 silicone cures in about 12 h. This constituted the elastic component of the SMH. The densities of E610 and E640 are 1.05 and 1.07 g/cm^3^, respectively. Since the difference is small, we used 1.06 g/cm^3^ for both of them in the calculations.

For the transition component, PCL (molecular weight: 60,000 g/mol) from Shenzhen Esun Industrial Co. Ltd. (Shenzhen, China) was used. The density of this PCL is 1.1 g/cm^3^. It is well known that, while the melting temperature of PCL is in the range of 60–70 °C, it can be crystallized at a body temperature of around 37 °C [48]. This characteristic is particularly useful for comfort fitting purposes as the user can program the shape to suit his/her needs at body temperature instead of at higher temperatures, where it might be too hot to handle comfortably.

To achieve porosity in the SMH samples, sodium polyacrylate hydrogel powders (from Tangshan Boya Shuzhi Co., Ltd., Tangshan, China) were used. The original size of these hydrogel powders ranges from 40 to 120 μm. These hydrogels can expand to around 500 times their original size upon exposure to water, and do not interact chemically with either silicone elastomer or PCL. Hence, the wetted hydrogels were used to introduce pores into SMH.

### 2.2. Samples Preparation

Porous samples were prepared with different volume percentages of silicone elastomer and PCL and with two different hydrogel sizes. All the porous samples have 30% wetted hydrogel by volume. In comparison, solid samples were also prepared. Refer to Table 1 for the composites of the samples prepared for this study.

For SMH sample preparation, initially equal amounts of Part A and Part B silicone elastomer were poured into two separate beakers and then, depending on the required volume ratio (30% or 40%), equal amounts of PCL pellets were added into them. For example, to prepare a sample with 60:40 ratio (60% E610 silicone and 40% PCL), 30 g of Part A was mixed with 21.5 g of PCL (calculated based on their densities) in a beaker. This mixture was heated to 100 °C for 20 min in an oven. Then, the mixture was retrieved from the oven and stirred vigorously for five minutes to ensure the uniform dispersion of PCL in the silicone. Subsequently, it was cooled down to room temperature of 25 °C. At the same time, a similar mixture was prepared with Part B instead of Part A. Once both mixtures were ready, they were mixed well at room temperature to obtain a uniform mixture. Then, 44 g of wetted hydrogel was added into this mixture and stirred thoroughly to obtain a uniform mixture. Here, wetted hydrogel prepared by wetting 1 g of dry hydrogel powder in 15 g of water is referred to as 50% wetting, whereas wetted hydrogel prepared by wetting of 1 g of dry hydrogel powder in 30 g of water is referred to as 100% wetting in this study.

Then, the mixture was poured into a cylindrical mould of 15 mm height and 50 mm diameter. The mould was placed at room temperature for around three hours to allow complete curing of the silicone. After curing, the sample was retrieved from the mould, and the wetted hydrogel was removed by hand squeezing. For pure silicone samples, similar fabrication steps were followed, but the only difference was that no PCL was added into either Part A or Part B. The same fabrication steps were followed for developing solid cylindrical SMH samples, as well as for the addition of the wetted hydrogel within them.

Table 2 shows the density and porosity of all types of samples. The weight of the samples was measured using a weighing scale with an accuracy of a decigram (0.1 g). The radius and thickness of the samples were measured using a vernier calliper with a least count of 0.05 mm.

Porosity was measured based on the ratio of volume of wetted hydrogel added during the fabrication process to the volume of the whole sample, i.e.,

(1) $$\mathrm{Porosity}=\frac{\mathrm{Volume}\;\mathrm{of}\;\mathrm{wetted}\;\mathrm{hydrogel}}{\mathrm{Volume}\;\mathrm{of}\;\mathrm{the}\;\mathrm{sample}}\times 100$$

Density was calculated by measuring the weight of the final sample (after removing the wetted hydrogel) and dividing it by the volume of the sample, i.e.,

(2) $$\mathrm{Density}=\frac{\mathrm{Weight}\;\mathrm{of}\;\mathrm{the}\;\mathrm{sample}}{\mathrm{Volume}\;\mathrm{of}\;\mathrm{the}\;\mathrm{sample}}$$

With the help of hydrogels during the fabrication process, the densities of these samples were reduced by about 27–33% which makes these samples lightweight and therefore usable in shoes. Even though the samples were prepared with 30% volume of wetted hydrogel, they show different porosity values, as seen in Table 2. This is because the wetted hydrogel particles had some water on their surface. This extra mass of water led to a slight difference in porosity.

**Figure 1:**
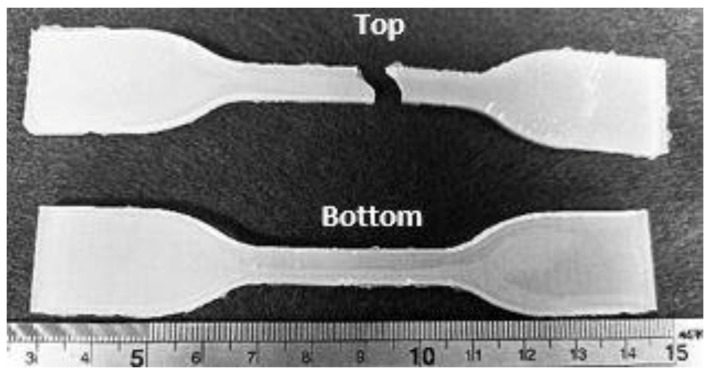
Original E640 sample (**bottom**) and stretched to fracture (**top**).

For the tensile test of pure silicone samples, the samples (3 mm thick) were prepared in a dog-bone shape following the ASTM D638 standard (type IV) procedure. Take the E640 dog-bone shaped sample (refer to Figure 1, bottom) as an example. Initially, equal amounts of Part A and Part B of E640 were uniformly mixed in a glass beaker for five minutes. Then, the uniform mixture was poured into the dog-bone mould and then left in the atmosphere for 15 min to eliminate air bubbles formed during stirring. Then, the top of the mould was closed to produce a uniformly thick sample. After curing at room temperature, the sample was taken out from the mould for testing.

**Figure 2:**
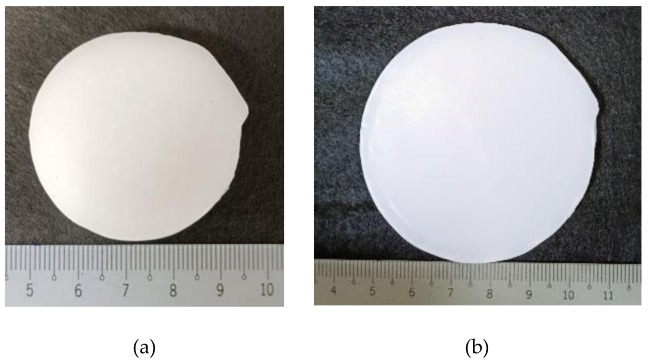
PCL sample (**a**) before and (**b**) after cyclic compression to 70%.

For the cyclic compression test of PCL, a cylindrical PCL sample was prepared with 4.6 cm diameter and 7 mm height (Figure 2a). The PCL sample was prepared by adding the PCL pellets into a cylindrical mould and then heated to 80 °C (above its melting point) in an oven for 20 min. Once all PCL pellets melted inside the mould, its retrieved from the oven and then cooled for crystallization. Finally, the cylindrical PCL sample was removed from the mould for testing.

### 2.3. Experimental Procedure

Differential scanning calorimeter (DSC) (TA DSC Q200 model, New Castle, DE, USA) was used to obtain the melting and crystallization temperatures of PCL. A small piece of sample about 10 mg was cut from the original pellet for testing. The sample was tested for two thermal cycles. In the first cycle, the sample was firstly cooled to −80 °C (for full crystallization) and then heated to 120 °C. Subsequently, the sample was cooled to 25 °C. In the second cycle, the sample was heated to 120 °C and then cooled to 25 °C. The applied heating/cooling speed was 10 °C/min.

Shore hardness (A) of solid samples was measured using SanLiang Shore hardness tester 324-211 LX-A (SanLiang, Dongguan, China). Pre-heated solid SMH samples were cooled by leaving them in the atmosphere at a room temperature of 25 °C. During this cooling process, the evolution of Shore hardness of these samples was continuously monitored at regular time periods. Parallelly, the surface temperatures of these samples were also recorded at the same time periods.

Unless otherwise stated, herein stress and strain denote engineering stress and engineering strain, respectively.

A Shimadzu AG-10kNXplus STD (Shimadzu Corporation, Kyoto, Japan) universal testing machine was used to perform uniaxial tensile test on pure silicone samples (both E610 and E640, dog-bone shape) till fracture, and all cyclic uniaxial compression tests on cylindrical samples. All these tests were conducted at room temperature. Unless otherwise stated, the applied strain rate was 10^−3^/s.

Cyclic uniaxial compression tests were performed to different compression strains of 10%, 20%, 30%, 40%, 50%, 60% and 70% in each cycle in incremental order. For some samples, which appeared to be much harder during testing, the test was stopped earlier before all cycles were completed because the force values exceeded the upper limit of the machine. To investigate the viscoelastic behaviour of pure silicone, both E610 and E620 (solid) were tested at two strain rates of 10^−2^/s and 10^−3^/s. Cyclic uniaxial compression tests were performed on original samples and SMH samples programmed with 20% or 40% compressive strain.

The cyclic uniaxial compression test was also carried out on pure PCL cylindrical sample to different compressive strains of 1%, 2%, 3%, 4%, 5%, 10%, 20%, 30%, 40%, 50%, 60% and 70%, in each cycle in incremental order.

A complete shape memory cycle, including programming and recovery processes, reveals the shape memory performance of a polymeric shape memory material [48]. The typical testing procedure is as follows.

1. A SMH cylindrical sample is heated to 80 °C, which is above the melting temperature of PCL for around 15 min in an oven. This process softens the PCL inclusions in the SMH which allows it to be easily deformed to a temporary shape.
2. The sample is retrieved from the oven and cooled down to body temperature of 37 °C.
3. The sample is compressed to a prescribed programming compressive strain ($\varepsilon_{m})$ in the above-mentioned universal testing machine at a strain rate of 10^−3^/s.
4. The sample is allowed to recrystallize by cooling it down to room temperature while maintaining the compressive strain for 30 min. The crystallization temperature of PCL used in this study is around 30 °C. This process hardens the PCL inclusions which in turn helps in fixing the temporary shape.
5. Unloading is carried out and the programmed sample is retrieved from the machine.

This completes the programming process of the shape memory cycle. The residual strain ($\varepsilon_{1})$ in the sample is noted. Creeping and relaxation are ignored in the course of this study [49].

Finally, the sample is heated to 80 °C for 15 min. This process once again softens the PCL inclusions and allows for the release of the stored elastic compressive energy inside the elastomer matrix which helps in shape recovery. The remaining strain ($\varepsilon_{2})$ is noted, and this is the end of the recovery process.

Shape fixity ratio (*R_f_*) and recovery ratio (*R_r_*) of the sample are calculated using the following formulae [48],

(3) $$R_{f}=\frac{\varepsilon_{1}}{\varepsilon_{m}}$$

(4) $$R_{r}=\frac{\varepsilon_{1}-\varepsilon_{2}}{\varepsilon_{1}}$$

In this study, three different programming compressive strains of 20%, 40% and 60% were used to analyse the shape memory performance of the porous SMH samples.

The distribution of pores was studied using Olympus SC30 optical microscope (Olympus Corporation, Tokyo, Japan) and the dispersion of PCL inclusions in the elastomer matrix was captured using the JSM 5000 scanning electron microscope (SEM) from JEOL, Ltd. (Tokyo, Japan). The original cylindrical sample (610:70:50) was cut in half and the cross-sectional surface was observed using the optical microscope to reveal the distribution of pores and using SEM to check the dispersion of PCL inclusions. Then, the other half piece of sample was programmed to 40% compressive strain at body temperature. The cross-sectional surface of this compressed piece was again observed using the optical microscope to see the distribution of pores and using SEM to reveal the dispersion of PCL inclusions.

To demonstrate the feasibility of body-temperature programming in real-life scenarios, the above programming process was utilized to capture a fingerprint on a solid rectangular SMH (60% E610 and 40% PCL) of 2 mm thickness, which was prepared in a similar way, as mentioned above. First, the rectangular SMH sample was heated to 80 °C for five minutes in an oven. Then, the sample was retrieved and allowed to cool down in air. The temperature was continuously monitored using a thermocouple. Once body temperature was reached, the fingerprint was captured by gently pressing the left thumb on the sample for about two minutes. In comparison, the same fingerprint was also captured on a wax sample when it was in a semi-liquid state. The TalyScan 150 instrument from Taylor Hobson (Leicester, UK) was used to capture the fingerprint profile.

In addition, the shape evolution of a porous sample (610:70:50) programmed by hand squeezing was investigated. The programming was performed by first heating the original sample to 80 °C for 15 min to soften the PCL inclusions. Then, it was cooled down to body temperature of 37 °C. At this temperature, the sample was hand squeezed and held in that shape till the PCL fully crystallized. Then, the deformed sample was recovered by heating it to 80 °C for 15 min.

## 3. Experimental Results and Analysis

**Figure 3:**
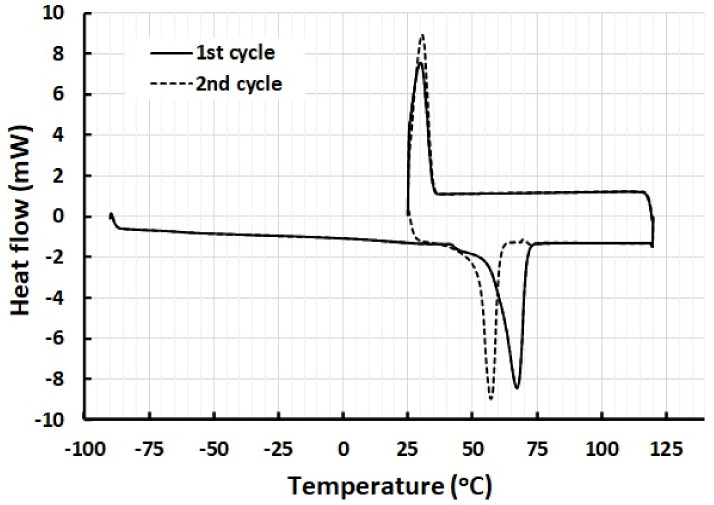
DSC result of PCL in two thermal cycles.

Figure 3 is the DSC result of PCL for two thermal cycles. The difference between the first and second cycles is mainly in the melting transition temperature range. The melting temperature decreases about 10 °C in the second heating process, while the crystallization temperature is always around 30 °C. Hence, upon heating to 80 °C, the PCL should fully melt, and programming can be done at body temperature.

**Figure 4:**
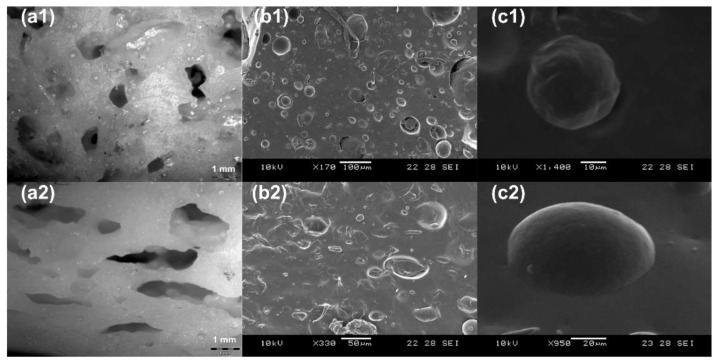
Pores and PCL inclusions in (610:70:50) sample. (**a1**): Pores in original sample (optical microscope); (**a2**): elliptical pores after programming (40% compression); (**b1**): circular PCL inclusions in original sample (SEM); (**b2**): elliptical PCL inclusions in 40% programmed sample; (**c1**): zoom-in view of one typical circular PCL inclusion in original sample (SEM); (**c2**): zoom-in view of one typical elliptical PCL inclusion in 40% programmed sample. Accelerating voltage of SEM: 5 kV.

Figure 4(a1) shows the distribution of almost circular pores with diameters ranging from 0.5 mm to 1.5 mm in 610:70:50 sample in its original shape. Many pores are actually connected. After programming the sample to 40% compressive strain, the pores became elliptical in shape (Figure 4(a2)). According to [13], if the volume fraction of the inclusion is more than 30%, the connection of the pores is likely to happen. Wetted hydrogel particles tend to aggregate because the adhesive force between wet hydrogel and silicone/PCL is very small. Therefore, wetted hydrogel particles do not mix uniformly within silicone/PCL. Consequently, hand squeezing can easily remove the wet hydrogels out of the prepared samples. Figure 4(b1,b2) provide details of the shape and dispersion of PCL inclusions in the sample before programming (circular shape with diameters ranging from 15 µm to 100 µm) and after programming to 40% compressive strain (elliptical shape with major axis diameters ranging from 25 µm to 120 µm), respectively. Figure 4(c1,c2) are the zoom-in views of a typical PCL inclusion in the silicone matrix before and after programming, respectively, which are similar to that reported in [38,41].

**Figure 5:**
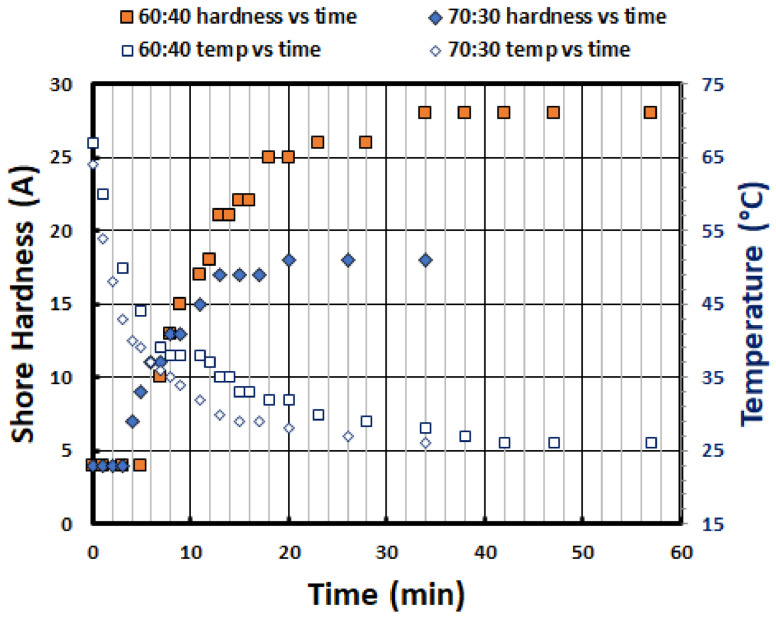
Evolution of shore hardness during cooling in the air for two solid SMH samples with 30% PCL and 40% PCL, respectively.

Figure 5 reveals the evolution of shore hardness of two solid SMH samples (one is with 60% silicone and 40% PCL, and the other is with 70% silicone and 30% PCL) during air cooling. The hardness vs. time curves for both SMH samples show that the samples only start to become harder after air cooling for four to five minutes from about 65 °C. The corresponding temperature is around body-temperature. Hence, we have an operating time of four to five minutes, before the samples are cooled to around body temperature in the air, for comfort fitting (programming) without rushing.

**Figure 6:**
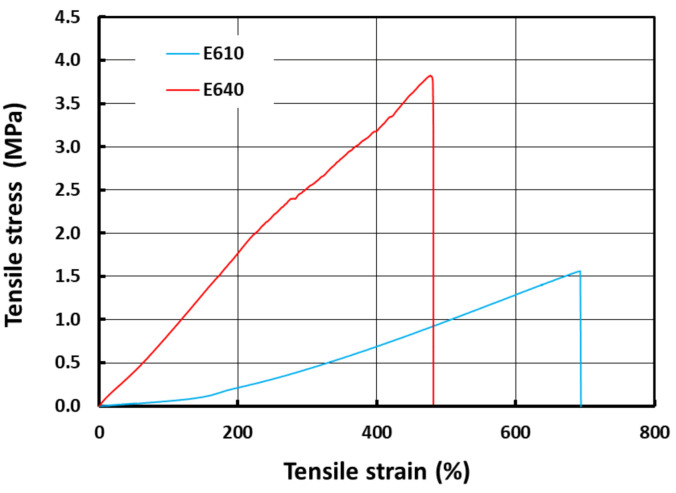
Stress vs. strain in uniaxial tension to fracture of pure silicones (E610 and E640).

Figure 6 compares the tensile stress vs. strain curve of dog-bone shaped samples made of pure E610 and E640, respectively. Both the samples easily achieve over 100% stretchability before fracture. In fact, E610 shows an extremely high elasticity and can be stretched to around 700% tensile strain before fracture. E640 also shows good elasticity where the fracture occurs after the application of around 400% tensile strain. Refer to Figure 1 for a comparison of the shapes of the original sample and fracture sample made of E640.

**Figure 7:**
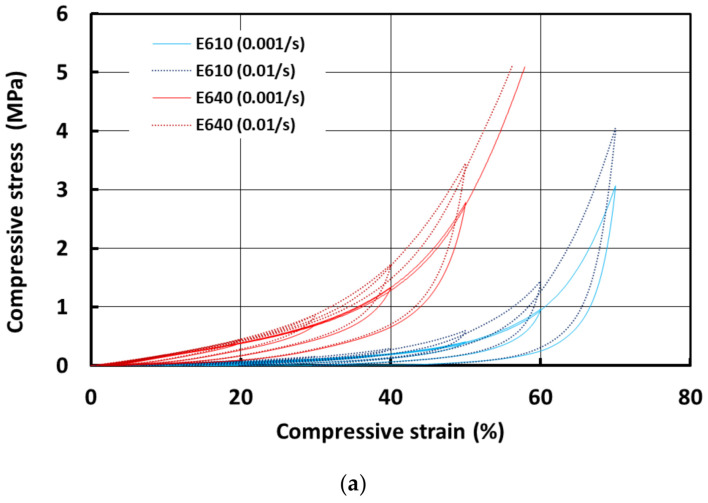

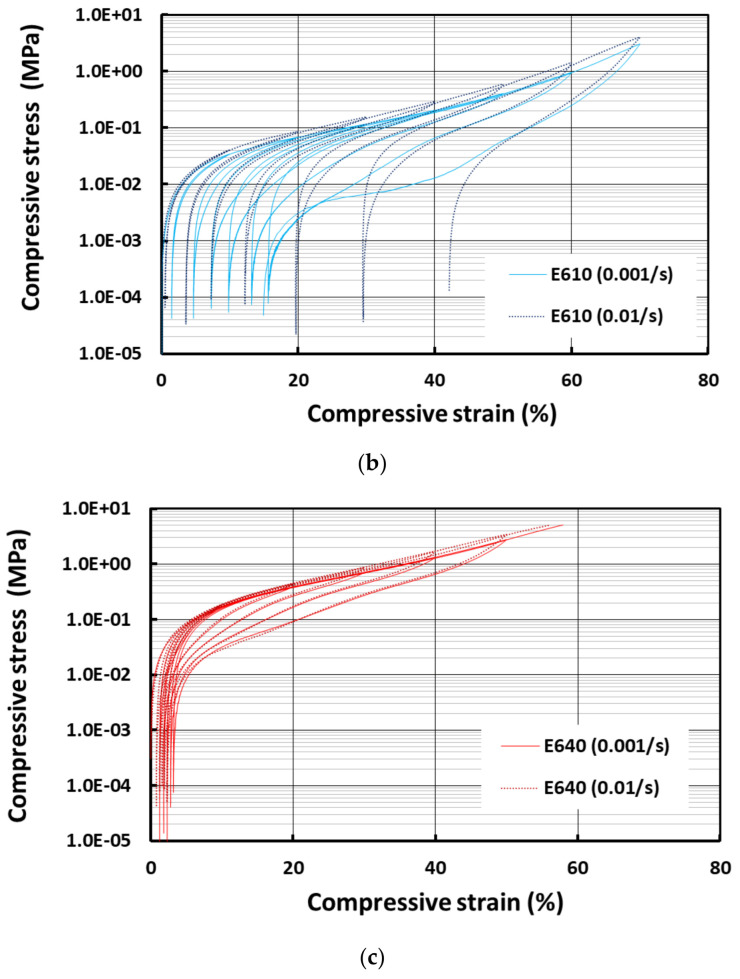
Influence of strain rate in cyclic uniaxial compression of pure silicones (E610 and E640). (**a**) Stress vs. strain curves at two different strain rates of 10^−2^/s and 10^−3^/s; (**b**) logarithmic plot of stress for E610; (**c**) logarithmic plot of stress for E640.

Figure 7a compares the stress vs. strain relationships of pure E610 and E640 (without pores) in cyclic uniaxial compression at two different strain rates of 10^−2^/s and 10^−3^/s. It appears that both silicones only show slight strain hardening effect. In order to have a better view of the residual strain in cyclic testing, the logarithmic plot of stress is used in Figure 7b,c for E610 and E640 samples, respectively. As we can see, the influence of strain rate on the residual strain upon unloading of E610 is far more significant than that of E640. Hence, in terms of the residual strain, E610 is more viscous, while E640 is more elastic. However, for the stress vs. strain relationship in the loading process, the influence of strain rate is not significant. Therefore, a constant strain rate of 10^−3^/s can be applied to compare the response of all samples in uniaxial compression at room temperature, since the compressive stress in loading is more of our interest.

**Figure 8:**
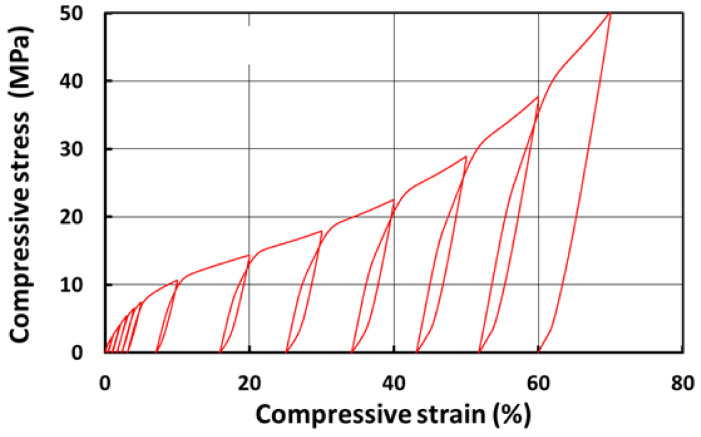
Stress vs. strain relationship of PCL in cyclic uniaxial compression at room temperature.

As shown in Figure 8, which is the stress vs. strain relationship of PCL in cyclic uniaxial compression at room temperature, this PCL is much harder than both silicones (E610 and E640). Hence, PCL inclusions are mostly able to maintain the shape during loading of SMH samples at room temperature.

**Figure 9:**
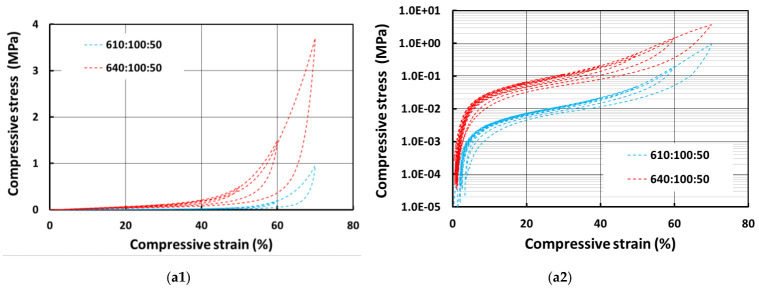

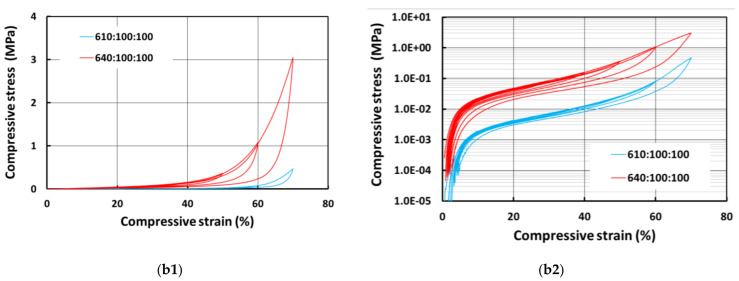
Stress vs. strain relationships of porous E610 and E640 samples prepared with 50% wetting hydrogel (**a1**), its corresponding logarithmic plot of stress in (**a2**) and 100% wetting hydrogel (**b1**) in cyclic uniaxial compression and its corresponding logarithmic plot of stress in (**b2**).

Figure 9 shows the compressive stress vs. strain curves of porous pure silicone samples prepared with 50% and 100% wetting hydrogel. The logarithmic plots (for stress) are also presented (right column). In comparison with the solid samples (Figure 7), as expected, porous samples are much softer. On the other hand, all porous samples appear to be less viscous. In particular, for the porous E610 sample, the residual strains are much lower when compared with that of the solid sample. The porous structure which helps to improve the elastic response, in particular if the compressive strain is not too high, should be the reason. A closer look reveals that the samples prepared with 100% wetting hydrogel are slightly softer than their counterparts that were prepared with 50% wetting hydrogel.

**Figure 10:**
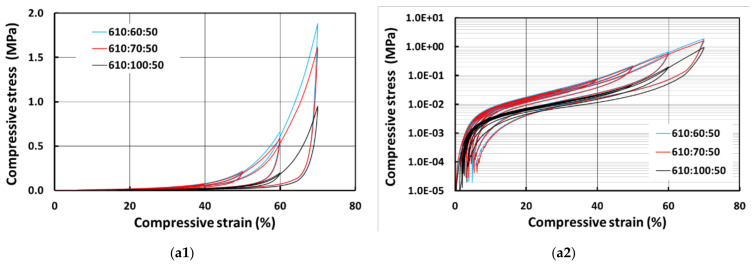

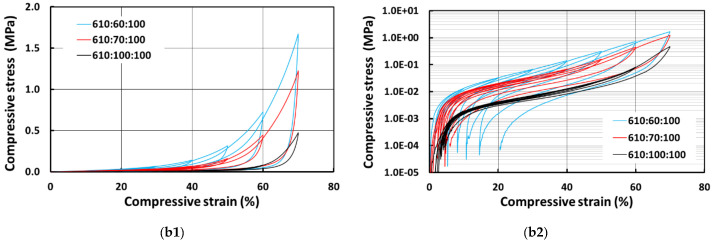
Compressive stress vs. strain curves for E610 samples prepared with 50% wetting hydrogel (**a1**), its corresponding logarithmic plot of stress in (**a2**) and 100% wetting hydrogel (**b2**) and its corresponding logarithmic plot of stress in (**b2**).

Figure 10 shows the compressive stress vs. strain curves for E610 samples prepared with 50% and 100% wetting hydrogel and their corresponding logarithmic plots on the right. As we can see, the samples with more PCL are harder, since PCL is much harder than E610 as revealed above. Again, the samples prepared with 100% wetting hydrogel are relative softer. An interesting finding is that while the residual strain in all samples prepared with 50% wetting hydrogel is still small, one of the samples prepared with 100% wetting hydrogel (610:60:100) has much larger residual strain.

**Figure 11:**
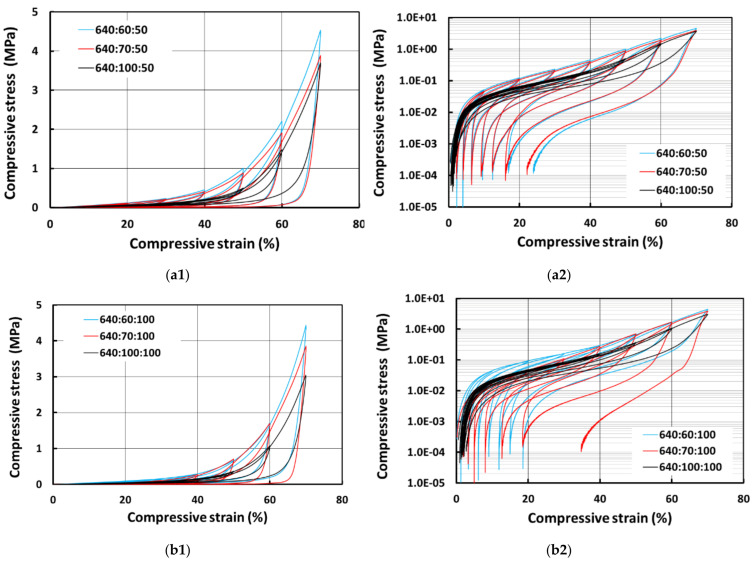
Compressive stress vs. strain curves for E640 samples prepared with 50% wetting hydrogel (**a1**), its corresponding logarithmic plot of stress in (**a2**) and 100% wetting hydrogel (**b2**) and its corresponding logarithmic plot of stress in (**b2**).

Figure 11 presents the results for E640 samples. The same trend can be observed as well, i.e., the samples with more PCL are harder, and the samples prepared with 100% wetting hydrogel are relative softer although the difference is smaller. The pure silicone samples show very low residual strains for both 50% and 100% wetting hydrogel. However, the samples with PCL and with 50% wetting hydrogel have a slightly higher residual strain of around 20% in the last cycle, whereas one of the samples with PCL and 100% wetting hydrogel (640:70:100) shows a much larger residual strain of around 37% in the last cycle.

**Figure 12:**
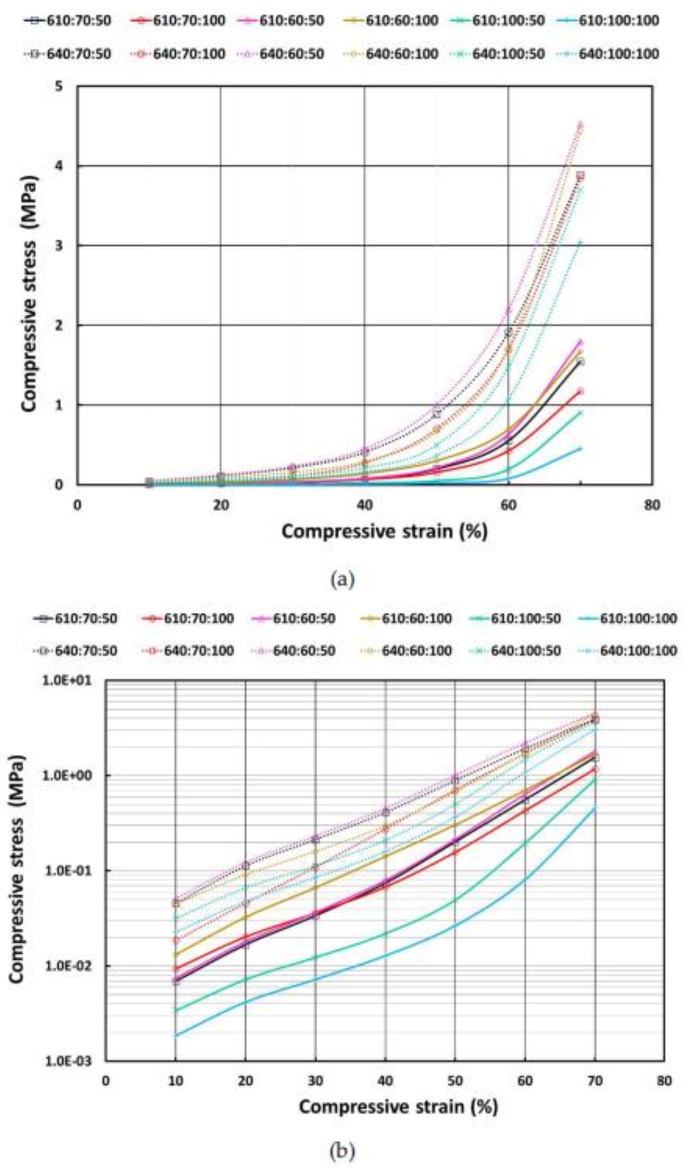
Stress at loading stop strain in cyclic compression of all porous samples (**a**); corresponding logarithmic plots (of stress) (**b**).

For easy comparison, during loading, the stresses corresponding to the prescribed programming strains in cyclic compression of all porous samples are plotted in Figure 12. Since E640 has a higher shore hardness of 40A, when compared to E610, which has only 10A hardness, any sample prepared with E640 has a higher compressive stress for a particular strain than a sample prepared with E610 and having the same amount of PCL and wetted hydrogel. PCL is an effective reinforcement material. Hence, with more PCL, the material becomes harder. The hydrogel size (via 50% or 100% wetting) also affects the strength of the material. One hundred percent wetting lowers the compressive stress.

**Figure 13:**
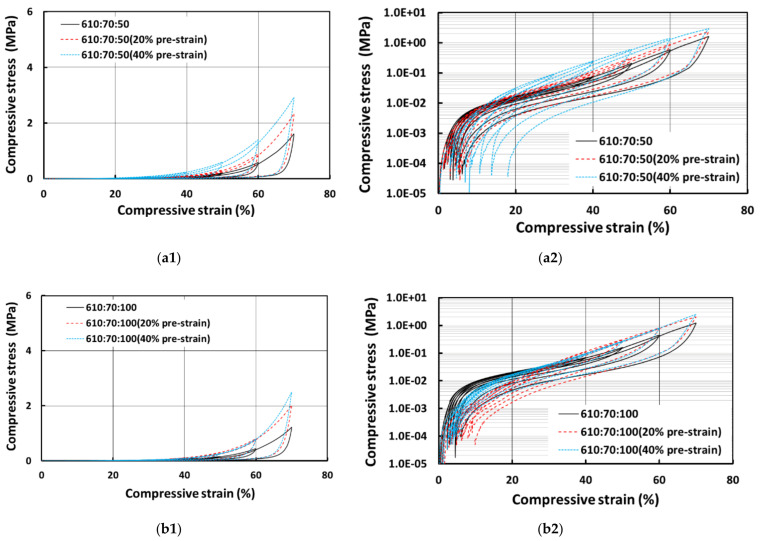

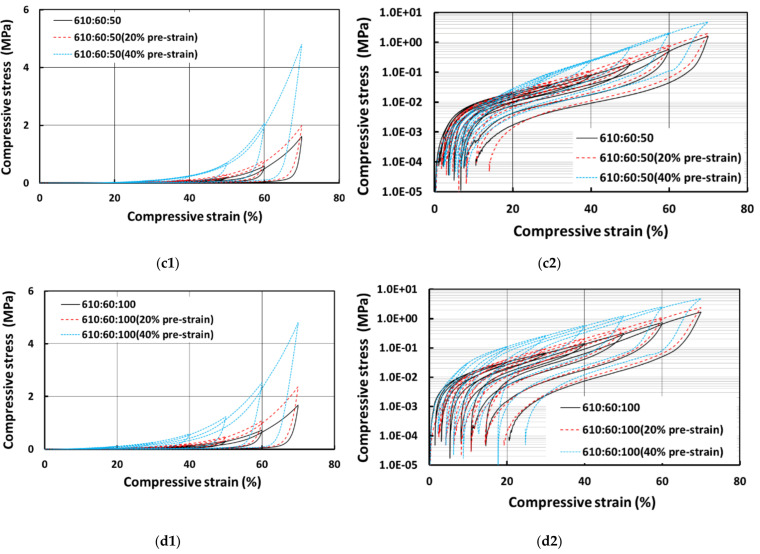
Compressive stress vs. strain of E610 SMH samples with 0%, 20% and 40% pre-strains; (**a1**) for samples with 70% silicone, 30% PCL and 50% wetting hydrogel and its corresponding logarithmic plot of stress in (**a2**); (**b1**) for samples with 70% silicone, 30% PCL and 100% wetting hydrogel and its corresponding logarithmic plot of stress in (**b2**); (**c1**) for samples with 60% silicone, 40% PCL and 50% wetting hydrogel and its corresponding logarithmic plot of stress in (**c2**); and (**d1**) for samples with 60% silicone, 40% PCL and 100% wetting hydrogel and its corresponding logarithmic plot of stress in (**d2**).

**Figure 14:**
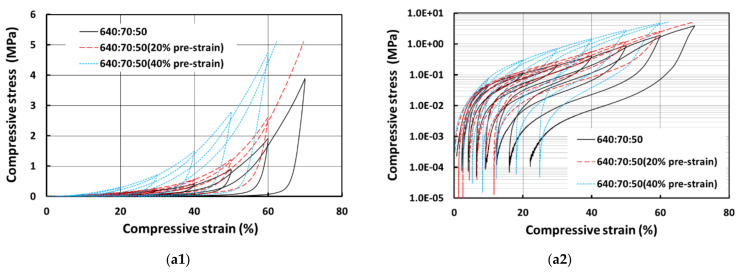

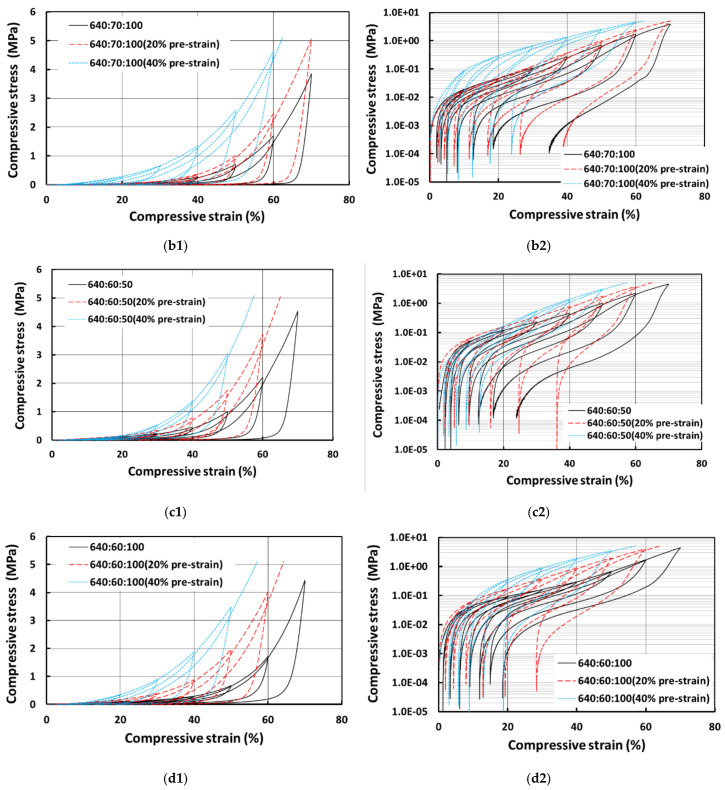
Compressive stress vs. strain of E640 SMH samples with 0%, 20% and 40% pre-strains; (**a1**) for samples with 70% silicone, 30% PCL and 50% wetting hydrogel and its corresponding logarithmic plot of stress in (**a2**); (**b1**) for samples with 70% silicone, 30% PCL and 100% wetting hydrogel and its corresponding logarithmic plot of stress in (**b2**); (**c1**) for samples with 60% silicone, 40% PCL and 50% wetting hydrogel and its corresponding logarithmic plot of stress in (**c2**); and (**d1**) for samples with 60% silicone, 40% PCL and 100% wetting hydrogel and its corresponding logarithmic plot of stress in (**d2**).

Figure 13 shows the comparison of SMH samples produced with E610 silicone at three different programming conditions. One is at original size, the second is programmed at 20% compressive strain (20% pre-strain) and the third is programmed at 40% compressive strain (40% pre-strain). The samples with highest pre-strain are much harder than samples without pre-strain as higher compression leads to increase in hardness. The same trend is also observed in Figure 14, which is for SMH samples produced with E640 silicone. However, for residual strain, we could not identify any particular trend. Some 40% pre-strained samples have the highest residual strains in the last cycle, whereas for some other cases, the samples programmed with 20% strain have the higher residual strains. Generally, the samples prepared with E640 silicone show slightly more residual strain than the samples prepared with E610 silicone.

It is worth noting that after the samples were retrieved from the machine, the residual strains gradually decreased, and the samples recovered their original shapes after an hour. This shows the viscoelastic behaviour of these samples. In addition to the viscoelasticity of the silicones, the interaction between PCL inclusions and the silicone matrix is the other major reason for gradual recovery. Since the bonding between PCL and silicone is not strong, similar to a silicone/melting glue SMH as reported in [44], debonding is easy to occur. Hence, the Mullins effect is observed [50]. The resulting friction between them dissipates more energy and slows down the speed of the recovery process.

**Figure 15:**
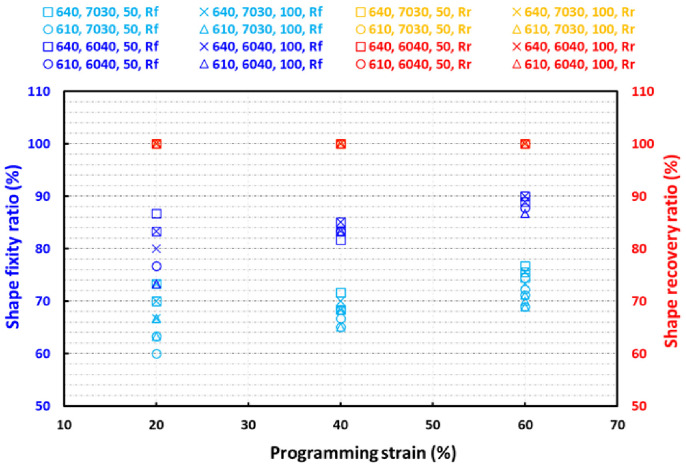
Shape fixity and shape recovery ratios of all samples.

Figure 15 shows the shape fixity and recovery ratios of all the samples at three different programming compressive strains of 20%, 40% and 60%, respectively. All the samples achieved full recovery upon the recovery process as observed by the value of 100% shape recovery ratios in all the samples in Figure 15. However, the shape fixity ratio depends on the amount of PCL in the sample and also depends on the applied programming compressive strain. The higher the amount of PCL, the higher the shape fixity ratio. This falls in line with the fact that higher amounts of inclusions (PCL) provide greater resistance to the elastic recovery of silicone elastomer in the SMH [38]. This is because PCL is much harder (about two orders of magnitude higher) than the silicone elastomers used in this study. Hence, higher amount of PCL resists higher amount of elastic compressive forces in the elastomer matrix. Consequently, a higher shape fixity ratio is achieved. Hence, the samples with higher amount of PCL tend to hold the programmed shape much better. The higher the programming compressive strain, the higher is the shape fixity ratio although the difference is small. One possible reason for this could be that the SMH sample is porous and so, for lesser programming compressive strains, the porous structure aids in recovering the shape slightly during the programming process. Hence, we see the shape fixity ratio to be slightly less for SMH samples programmed at 20% compressive strain than for samples programmed at higher compressive strains. The type of silicone does not affect the shape fixity ratio in a significant manner, as both silicones used in this study are much softer than the inclusions (PCL). The hydrogel size (via 50% or 100% wetting) only has slight effect on the shape fixity ratios of the SMH.

We achieved a maximum of 90% shape fixity ratio for samples with 40% PCL inclusions in them. Achieving a higher shape fixity ratio is difficult because of the following reasons [38]. As explained, PCL is responsible for shape fixing. The higher the percentage of PCL in the SMH, higher is the shape fixity ratio. However, over 40% PCL in the SMH would lead to aggregation of PCL inclusions within the elastomer matrix and the final SMH would not be uniform and consistent. Moreover, the programming was done at body temperature, where it is possible that some PCL inclusions would have already crystallized before the programming started. Hence, achieving a shape fixity ratio of more than 90% is practically difficult.

**Figure 16:**
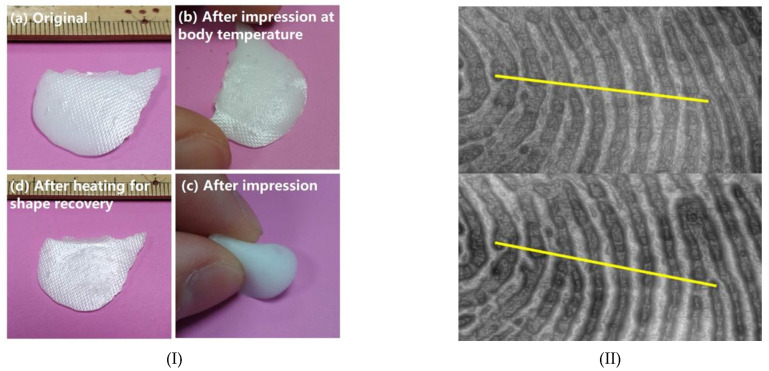
Fingerprint capture in a solid SMH (60:40 of silicone:PCL) (**I**); and comparison with wax (**II**) (top: silicone/PCL; bottom: wax).

The shape memory performance of a typical solid SMH (with 60% E610 and 40% PCL) was demonstrated by capturing a fingerprint on its surface and then comparing it to an identical fingerprint captured on a wax surface, which is able to precisely capture the original fingerprint. Figure 16I shows the SMH sample at four different stages. Figure 16(Ia) is the original sample (with surface pattern). After programming at body temperature, we can clearly see a fingerprint on the surface (Figure 16(Ib)). Since the material is highly elastic, deformation, such as severe bending, as shown in Figure 16(Ic), does not destroy the captured fingerprint. After heating for shape recovery, the original surface pattern returns (Figure 16(Id)).

**Figure 17:**
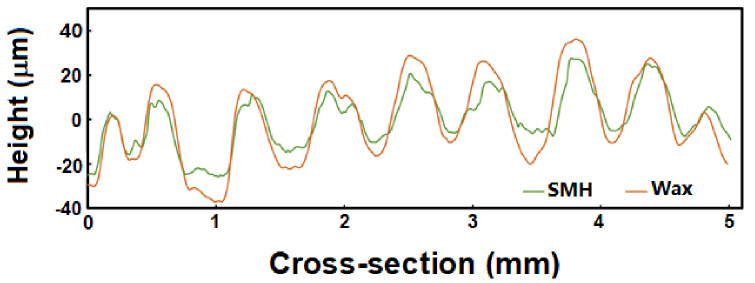
Cross-section profiles of wax and SMH samples.

In Figure 16II, we compare the fingerprints captured by the SMH sample (top) and wax (bottom). It can be seen that the contours of the fingerprint captured on the SMH are consistent with that of the wax sample. To further compare the precision of the fingerprints captured by the SMH sample and wax, we examined the same cross-section (as marked in Figure 16II) of both captured fingerprints to compare the depth profile. It is worth noting that it is very hard to get the exact same fingerprint (in every detail) every time. Hence, based on the results in Figure 17, we can claim that the SMH sample is able to capture the fingerprint profile almost exactly as that of the wax.

**Figure 18:**
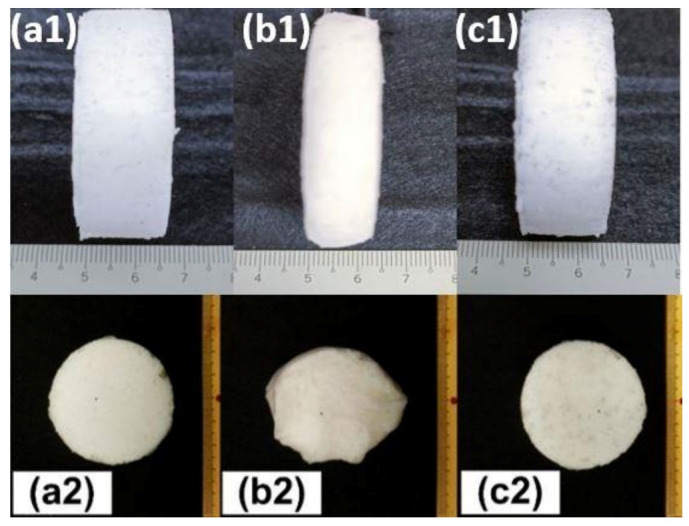
Shape evolution. (**a1**) original sample, (**b1**) programmed after compression to 40% strain and (**c1**) after recovery; (**a2**) original sample, (**b2**) programmed by hand squeezing and (**c2**) after recovery.

Figure 18 (top row) shows the evolution in height of a cylindrical SMH (610:70:50) when it was compressed by 40%. Full height recovery is observed after heating. Figure 18 (bottom row, pictures taken from top view) shows the shape evolution of the same porous sample (610:70:50) programmed by hand squeezing at body temperature (Figure 18(a2)). Then, the deformed sample (Figure 18(b2)) was recovered by heating it to 80 °C for 15 min. Good shape recovery is revealed in Figure 18(c2).

## 4. Shape Memory Soles and Shape Memory Sock-Shoes

**Figure 19:**
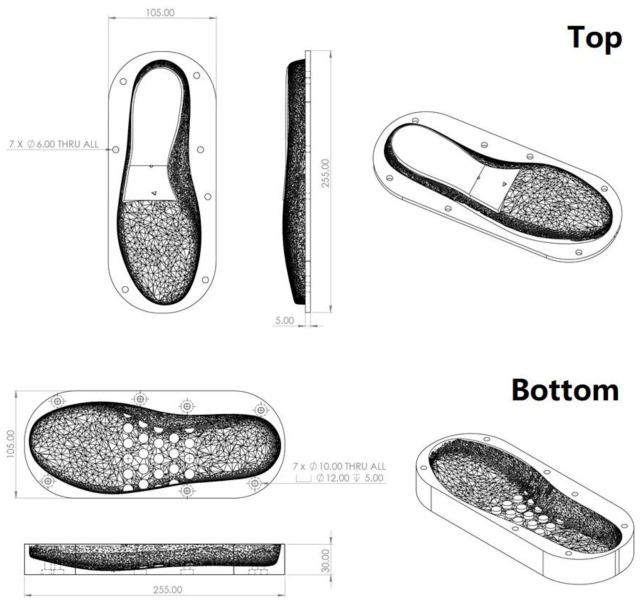
Dimensions of moulds used to produce SMH sole (size: 37.5; left side).

Shape memory soles were developed using the moulds as shown in Figure 19 (for left side 37.5 sized shoes, including the top and bottom two parts). The moulds were 3D printed using 1.75 mm diameter PLA filament (bought from Shenzhen Esun Industrial Co. Ltd., Shenzhen, China) via fused deposition modelling (FDM) (Creality CR 10, Shenzhen, China).

**Figure 20:**
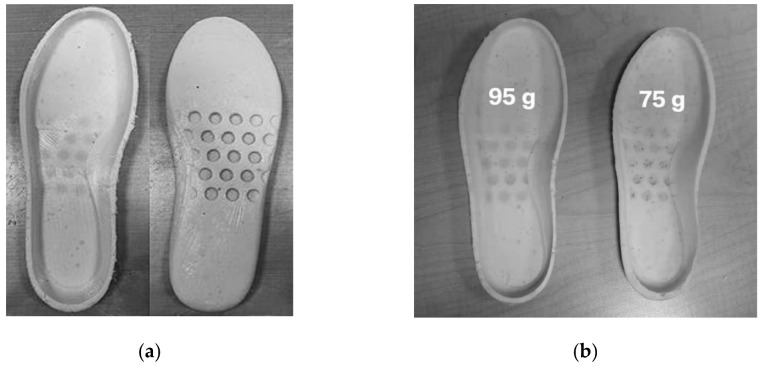
Shape memory soles. (**a**) Top and bottom sides of solid shape memory sole (120 g); (**b**) two pieces of shape memory sponge soles. Right: 75 g and left: 95 g.

Using these moulds, solid and sponge shape memory soles were fabricated (Figure 20). The weight of the solid sole (Figure 20a) was 120 g, and two sponge soles (Figure 20b) were measured to be around 95 g (left) and 75 g (right). A clear weight reduction of around 21% and 37%, respectively, were achieved for the sponge soles.

**Figure 21:**
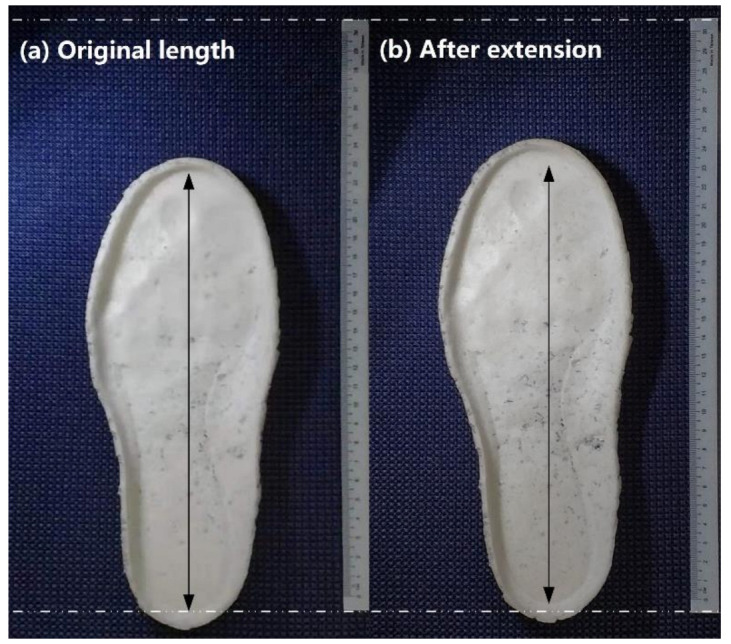
Body-temperature programming of shape memory sponge sole. (**a**) After body-temperature stepping on it (footprint captured); (**b**) after body-temperature stretching.

The fabricated soles were then tested for their shape memory performance. Two tests were performed. The first test involved heating the whole sole and then stepping on it at body-temperature. As shown in Figure 21a, footprints were clearly captured. The second test involved heating the middle part of the sole and stretching it slightly at body temperature. As revealed in Figure 21b, the sole became 1 cm longer.

**Figure 22:**
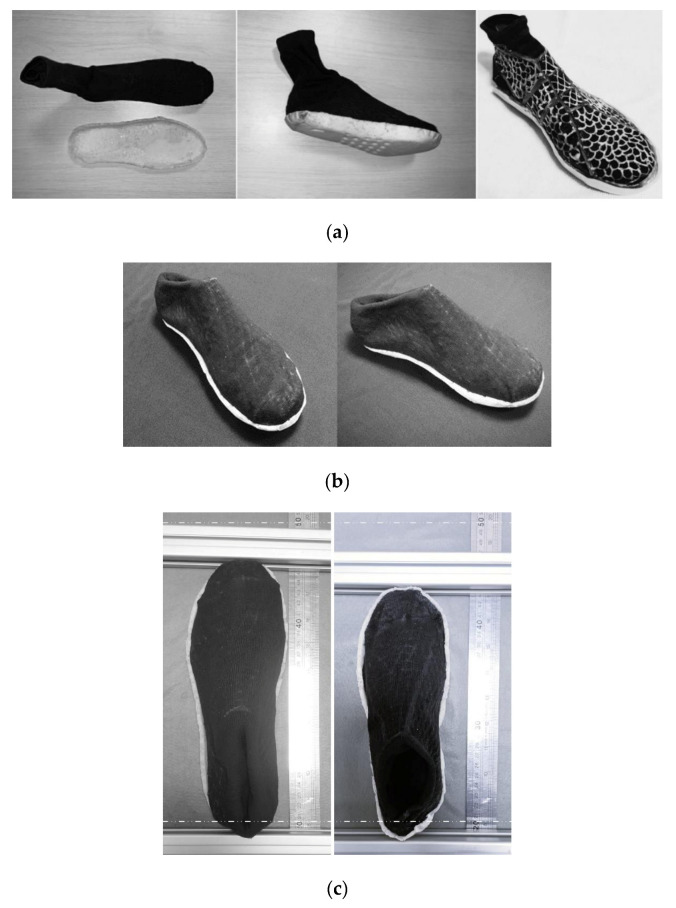
Shape memory sock-shoes. (**a**) Left: before assembly; middle and right: two versions of sock-shoes; (**b**) after fitting of Figure 22a middle image; (**c**) comparison of size (left: after fitting; right: after recovery).

Using these materials, a sock-shoe was fabricated following the steps described below. PCL mesh was printed on top of a sock with a shoe-last inside via a 3D printing pen as shown in Figure 22a (rightmost picture). This sock with PCL mesh was inserted into another sock and heated at 80 °C for five minutes to melt the PCL and then compressed to bond the two socks together (Figure 22a, leftmost image).

Silicone glue (4120, Shenzhen Hong Ye Jie Technology Co Ltd., Shenzhen, China) was used as the bonding agent to bond the shape memory sponge sole to this sock. The silicone glue also comes in two parts, A and B. They were mixed in a 1:1 ratio, as recommended by the manufacturer. A thin layer of this mixture was applied to bond the sole uniformly at the bottom of the sock. The silicone glue fully cured in two days at room temperature.

The fabricated sock-shoe is shown in Figure 22a (middle image).

After fabrication, the shoe was heated to 80 °C for ten minutes and then cooled down to body temperature. Then, a volunteer with a foot size of 40.5 stepped into the shoe and wore it until the PCL crystallized. The programmed sock-shoe images are shown in Figure 22b. Figure 22c shows the comparison of the original sock-shoe (right, size: 37.5) and the programmed sock-shoe (left, size: 40.5). The weight of the sock-shoe is 140.6 g, which is considerably less than the weight of the running shoe of an average male, which is about 200 g.

## 5. Conclusions

Soft, lightweight and eco-friendly SMH samples with different amounts of PCL were fabricated using wetted hydrogel. Apart from being soft, it was observed that (a) weight reduction of around 30% was achieved by using wetted hydrogel in the fabrication process and further reduction is also possible; (b) the produced samples exhibited excellent elasticity; (c) higher PCL content produces harder samples; and (d) for a fixed porosity of about 30%, the hardness of SMH samples can be tailored by selecting a silicone with different hardness and with different amount of PCL.

All SMH samples showed 100% shape recovery ratio. SMHs with 40% PCL exhibited a higher shape fixity ratio of around 90%, thereby making them excellent materials for footwear with very good comfort-fitting features. SMH samples with E610 silicone (shore hardness: 10A) are extremely soft which helps in improving the comfort levels of the footwear. E640 (shore hardness: 40A) offers a much harder and sturdier variant that can withstand higher amount of stress. The PCL in the SMHs hardens at around 30 °C and crystallizes in about three to four minutes by air cooling at room temperature. Hence, these SMHs can be programmed at body temperature with enough time for fitting. This property combined with excellent shape memory performance makes these shape memory hybrids extremely viable materials that can be used, for instance as soles, for comfort fitting purposes.

## Figures and Tables

**Table 1:** Sponge samples.

Sample		Silicone Elastomer	Silicone (vol%)	PCL (vol%)	Hydrogel Wetness
1	610:70:50	E610	70	30	50%
2	610:70:100	E610	70	30	100%
3	610:60:50	E610	60	40	50%
4	610:60:100	E610	60	40	100%
5	610:100:50	E610	100	0	50%
6	610:100:100	E610	100	0	100%
7	640:70:50	E640	70	30	50%
8	640:70:100	E640	70	30	100%
9	640:60:50	E640	60	40	50%
10	640:60:100	E640	60	40	100%
11	640:100:50	E640	100	0	50%
12	640:100:100	E640	100	0	100%

**Table 2:** Densities and porosities of the samples.

Sample		Weight (g)	Volume (cm^3^)	Porosity (%)	Density (g/cm^3^)
1	610:70:50	21.6	29.44	29.89	0.73
2	610:70:100	21.7	29.44	29.89	0.74
3	610:60:50	22.8	30.42	28.93	0.75
4	610:60:100	22.7	30.42	28.93	0.75
5	610:100:50	19.4	27.33	32.20	0.71
6	610:100:100	19.1	27.71	31.76	0.69
7	640:70:50	21.6	29.05	30.30	0.74
8	640:70:100	21.6	29.44	29.89	0.73
9	640:60:50	22.3	29.44	29.89	0.76
10	640:60:100	21.5	28.46	30.92	0.76
11	640:100:50	22	29.83	29.50	0.74
12	640:100:100	21.8	29.55	29.78	0.74
13	610:70:0	32.6	30.22	0	1.08
14	610:60:0	33.1	30.51	0	1.08
15	610:100:0	32.5	30.81	0	1.05
16	640:70:0	32	29.44	0	1.09
17	640:60:0	32.3	29.59	0	1.09
18	640:100:0	31.6	29.44	0	1.07
